# Mechanistic and Kinetic Analysis of Na_2_SO_4_-Modified Laterite Decomposition by Thermogravimetry Coupled with Mass Spectrometry

**DOI:** 10.1371/journal.pone.0157369

**Published:** 2016-06-22

**Authors:** Song Yang, Wenguang Du, Pengzheng Shi, Ju Shangguan, Shoujun Liu, Changhai Zhou, Peng Chen, Qian Zhang, Huiling Fan

**Affiliations:** 1 Key Laboratory for Coal Science and Technology of Ministry of Education and Shanxi Province, Institute for Chemical Engineering of Coal, Taiyuan University of Technology, Taiyuan 030024, China; 2 College of Chemistry and Chemical Engineering, Taiyuan University of Technology, Taiyuan 030024, China; Southwest University, CHINA

## Abstract

Nickel laterites cannot be effectively used in physical methods because of their poor crystallinity and fine grain size. Na_2_SO_4_ is the most efficient additive for grade enrichment and Ni recovery. However, how Na_2_SO_4_ affects the selective reduction of laterite ores has not been clearly investigated. This study investigated the decomposition of laterite with and without the addition of Na_2_SO_4_ in an argon atmosphere using thermogravimetry coupled with mass spectrometry (TG-MS). Approximately 25 mg of samples with 20 wt% Na_2_SO_4_ was pyrolyzed under a 100 ml/min Ar flow at a heating rate of 10°C/min from room temperature to 1300°C. The kinetic study was based on derivative thermogravimetric (DTG) curves. The evolution of the pyrolysis gas composition was detected by mass spectrometry, and the decomposition products were analyzed by X-ray diffraction (XRD). The decomposition behavior of laterite with the addition of Na_2_SO_4_ was similar to that of pure laterite below 800°C during the first three stages. However, in the fourth stage, the dolomite decomposed at 897°C, which is approximately 200°C lower than the decomposition of pure laterite. In the last stage, the laterite decomposed and emitted SO_2_ in the presence of Na_2_SO_4_ with an activation energy of 91.37 kJ/mol. The decomposition of laterite with and without the addition of Na_2_SO_4_ can be described by one first-order reaction. Moreover, the use of Na_2_SO_4_ as the modification agent can reduce the activation energy of laterite decomposition; thus, the reaction rate can be accelerated, and the reaction temperature can be markedly reduced.

## Introduction

Nickel has good plasticity, corrosion resistance and magnetic properties and is widely used in the iron and steel, nickel-based alloy electroplating, and the battery industries. These uses have led to a dramatic increase in the production of nickel in recent years [1]. Nickel can be obtained from nickel sulfide ores and nickel laterite ores. Although nickel sulfide ores can be treated to make them easier to process to obtain high grade nickel and improve recovery, they are becoming increasingly rare. Therefore, nickel laterite, which composes more than 70% of nickel resources and is easy to mine and transport, has attracted large amounts of attention in recent years and will be the main source of nickel in the future [2].

Nickel laterite ores can be divided into two different types based on their chemical and physical characteristics: saprolitic and limonitic ores [3]. Saprolitic ores contain a heterogeneous distribution of quartz, talc, serpentine, olivine, garnierite and high-grade nickel. Limonitic ores are buried near the ground surface and contain goethite, gibbsite, and chromite [4,5]. However, these nickel laterites cannot be effectively used in physical methods because of their poor crystallinity and fine grain size. Moreover, nickel cannot be identified in laterites by normal X-ray diffraction (XRD) detectors because of their low Ni concentrations and variable distributions. Goethite and silicate minerals (lizardite and olivine) are the Ni host phases [6]. Therefore, effectively upgrading these ores by physical treatment processes can be challenging. To solve this problem, pyrometallurgical production methods have been applied to extract nickel from laterite ores, which can obtain high nickel recoveries [7]. However, because of their high energy consumption and the low accumulation rate of Ni, the use of these methods is limited by low-grade laterite ores and extremely high operating temperatures [8]. Therefore, pre-reduction followed by magnetic separation has been proposed as an easy, low-energy-consumption and environmentally friendly process [9,10]. Laterite contains large amounts of water, which includes water that is adsorbed on the crystals and water in the mineral structure [1,7]. Water makes up 35% of the total quantity of the laterite; thus, laterite must be dried before the pre-reduction process. Moreover, the complex mineral composition of laterite will strongly affect the reduction roasting process. Therefore, studying the decomposition of laterite is important for the pre-reduction of laterite.

Recently, several researchers have focused on the reductive roasting of laterite ore with different reductants, such as carbon, CO or H_2_, followed by magnetic separation [9,10]. Reductive roasting of laterite can provide high nickel recovery, but the grade of the nickel remains low. Because nickel is hosted in goethite and silicate minerals, something must be added to break the bonds of the silicate and nickel; the nickel can then be easily reduced by a reductant. Therefore, additives such as Na_2_CO_3_, S, NaCl and Na_2_SO_4_ are used to enhance the enrichment ratio of Ni and reduce the reaction temperature [11,12]. Na_2_SO_4_ is the most efficient additive in terms of both grade enrichment and the recovery of Ni. The addition of 10–20 wt% Na_2_SO_4_ and 2% wt% coal, which was reduced at 1100–1200°C, resulted in a Ni content of 9.87% and a nickel recovery of 90.90% [11,12]. The Na_2_SO_4_ has two main effects: forming low-melting-point FeS to aggregate the ferronickel particles and suppressing the reduction of the ferrous minerals [13]. However, exactly how Na_2_SO_4_ reacts with laterite and when the reaction occurs has not been clearly investigated. Considering the potential application of Na_2_SO_4_ as a modification agent, the optimal operational conditions of the system should be determined. Based on the discussion presented above, this study investigated the decomposition characteristics of laterite with and without the addition of Na_2_SO_4_ using thermogravimetry coupled with mass spectrometry (TG-MS) to explore the effect of Na_2_SO_4_ on laterite decomposition. To further explore the reduction-promoting mechanisms of Na_2_SO_4_ on laterite ores, we also performed reduction roasting experiments of laterite ores with and without Na_2_SO_4_ at different roasting temperature points. The results of this study may provide a reference for the application of additives in related industries.

## Materials and Methods

### Materials

The feedstock material that was used in this study was a low-grade laterite ore from Indonesia. The as-received sample was ground to a particle size of less than 0.83 mm. Reagent-grade sodium sulfate was selected as the modification reagent.

An inductively coupled plasma atomic emission spectrometer (ICP-AES-9000(N+M)), which was a commercial product of Thermo Jarrell-Ash Corp., USA, was used to determine the chemical composition of the materials. A chemical phase analysis was performed to identify the distribution of nickel in the laterite; the results are listed in Table 1.

**Table 1:** The main chemicals and nickel distribution of the studied laterite (wt%).

	Nickel distribution state of the studied laterite ore								
Ni_total_	Adsorption			Sulfides		Oxides		Silicates	
1.41	0.03			0.07		0.12		1.19	
Fe_total_	Cr_2_O_3_	Al_2_O_3_	CaO	MgO	SiO_2_	P_2_O_5_	MnO_2_	Co	LOI^a^
24.14	1.08	3.15	1.46	14.58	29.36	0.018	1.46	0.065	12.95

^a^LOI: Loss on ignition.

Table 1 shows the main chemicals and the nickel distribution of the studied laterite, which contained 1.41 wt% Ni, 24.14 wt% Fe, 14.58 wt% MgO, 29.36 wt% SiO_2_ and 3.15 wt% Al_2_O_3_ and thus was a typical saprolite laterite [14]. The nickel was mainly hosted in silicate minerals (84.40%).

## Methods

### Samples

The laterite ore was mixed with sodium sulfate by mechanical stirring. The content of the sodium sulfate was 20 wt%, and the corresponding samples were denoted by Na_2_SO_4_/laterite blend ores. Laterite ore without Na_2_SO_4_ was used as the control.

### Laterite decomposition

The decomposition of the laterite was carried out using a Setaram SETSYS TGA coupled with a Hiden HPR20 QIC R&D mass spectrometer. Approximately 25 mg of the sample was pyrolyzed under a 100 ml/min Ar flow at a heating rate of 10°C/min from room temperature to 1300°C. The mass loss (TG) and the derivative thermogravimetric (DTG) curves with the temperature were obtained from the results of the experiment [15]. The kinetic study was based on the derivative DTG curves. The evolved gaseous compounds that were generated during the decomposition of the laterite were detected by MS. The different crystalline phases of the ores were determined by XRD using Cu-Kα radiation with a scanning rate of 3°/min from 5°–85° [15].

To ensure the uniformity of the sample and considering the small amount needed for the TG test, three sets of duplicate experiments were performed.

### Decomposition of the Na_2_SO_4_/laterite blends

The samples of the Na_2_SO_4_/laterite blends were subjected to pyrolysis following a process similar to that described in the preceding subsection. MS and XRD were performed.

Three sets of duplicate experiments were performed for the TG test.

### Kinetic analysis

The kinetic parameters were determined by the integral method, which assumed that the decomposition of laterite with and without the addition of Na_2_SO_4_ occurred in multiple stages and is a first-order reaction for each stage [16–18]. The following results indicate that this assumption is reasonable. The rate reactions of the solid-state can be then expressed using differential kinetic equations [19–20]. The decomposition reaction of laterite with and without the addition of Na_2_SO_4_ was obtained from the literature [18] and is expressed by the following formula:

$$\frac{d\alpha }{dt}=\text{k}(\text{t})\cdot f(\alpha )$$ (1)

where *α* is the decomposition conversion, *t* is time, k(t) is the temperature-dependent rate constant, and *f*(*α*) is a function that represents the reaction model.

The decomposition conversion, *α*, can be expressed by [18]

$$\alpha =\frac{W_{0}-W_{t}}{W_{0}-W_{f}}$$ (2)

where *W*_*0*_ is the original weight of the test sample, *W*_*t*_ is the weight at time t, and *W*_*f*_ is the final weight at the end of the decomposition.

Generally, k(t) is written as an Arrhenius relation [21]:

$$\text{k}(\text{t})=A\exp\left(-\frac{E}{RT}\right)f(\alpha )$$ (3)

where *E* is the activation energy, *A* is the pre-exponential factor, and *T* is the temperature.

Because *Θ = dT/dt*, which is a constant heating rate, we can rearrange and integrate Eq (3); the integration can then be expressed as follows [22]:

$$\text{ln}\left[\frac{\text{-ln}(1-\alpha )}{T^{2}}\right]=ln\left[\frac{AR}{\Theta E}\left(1-\frac{2RT}{E}\right)\right]-\frac{E}{RT}$$ (4)

Because *Θ* is a constant (10°C/min) during decomposition and the temperature range of laterite decomposition is much larger than *2RT* for most values of *E*, the expression *2RT/E* is nearly equal to 0 [16]. Therefore, Eq (4) can be rearranged as follows [19]:

$$\text{ln}\left[\frac{\text{-ln}(1-\alpha )}{T^{2}}\right]=ln\left(\frac{AR}{10E}\right)-\frac{E}{RT}$$ (5)

The straight line that can be obtained from the left side of Eq (5) is plotted versus 1/T because the process can be assumed to be a first-order reaction.

The evolutions of the weight and weight loss rate with increasing temperature were obtained for the decomposition. The weight loss rate was calculated by the following expression [23]:

$$\frac{dW}{dt}=-\frac{1}{W_{0}}(\frac{dWt}{dt})$$ (6)

where *W*_*0*_ is the original weight of the test sample, and *W*_*t*_ is the weight at time *t*.

### Reduction experiments

Laterite with 20% Na_2_SO_4_ with a combined mass of 100 g was prepared for the reduction process, which was performed in a stirred fixed-bed reactor [9]. After the samples were heated to the determined temperature (700, 800, or 900°C) under a nitrogen flow rate of 0.7 L/min, a reaction was achieved by mixing gas (70% H_2_ and 30% N_2_) at 2.7 L/min for 120 min. The N_2_ was used as a protective gas as the reduction process completed until the samples cooled to room temperature.

The reduced samples were ground to 90 wt% passing 0.043 mm using a rod mill. Then, approximately 5 g of each ground sample was separated in an XCGS-73 Davies magnetic tube with a magnetic field intensity of 0.1 T [7]. The final magnetic product consisted of FeNi concentrates. The content of Fe and Ni in each sample was determined by chemical analysis. The recovery rate of Fe and Ni was calculated as described in the literature [9].

The reduced products were analyzed using scanning electron microscopy (SEM; Carl Zeiss EVO18, Germany) according to the literature [11].

## Results and Discussion

### Decomposition of laterite with and without the addition of Na_2_SO_4_

**Fig 1:**
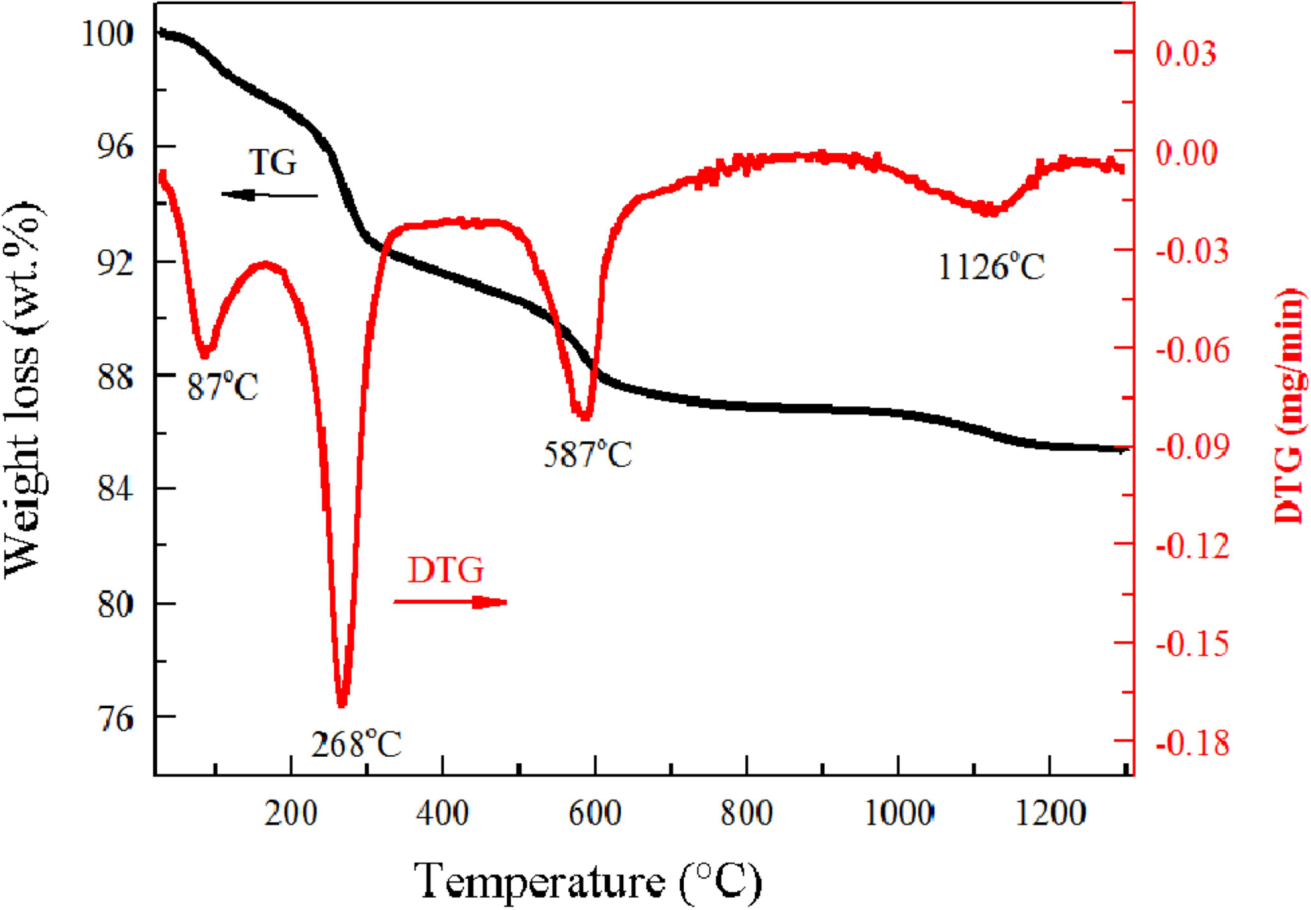
Differential thermal analysis–TGA curves for laterite.

**Fig 2:**
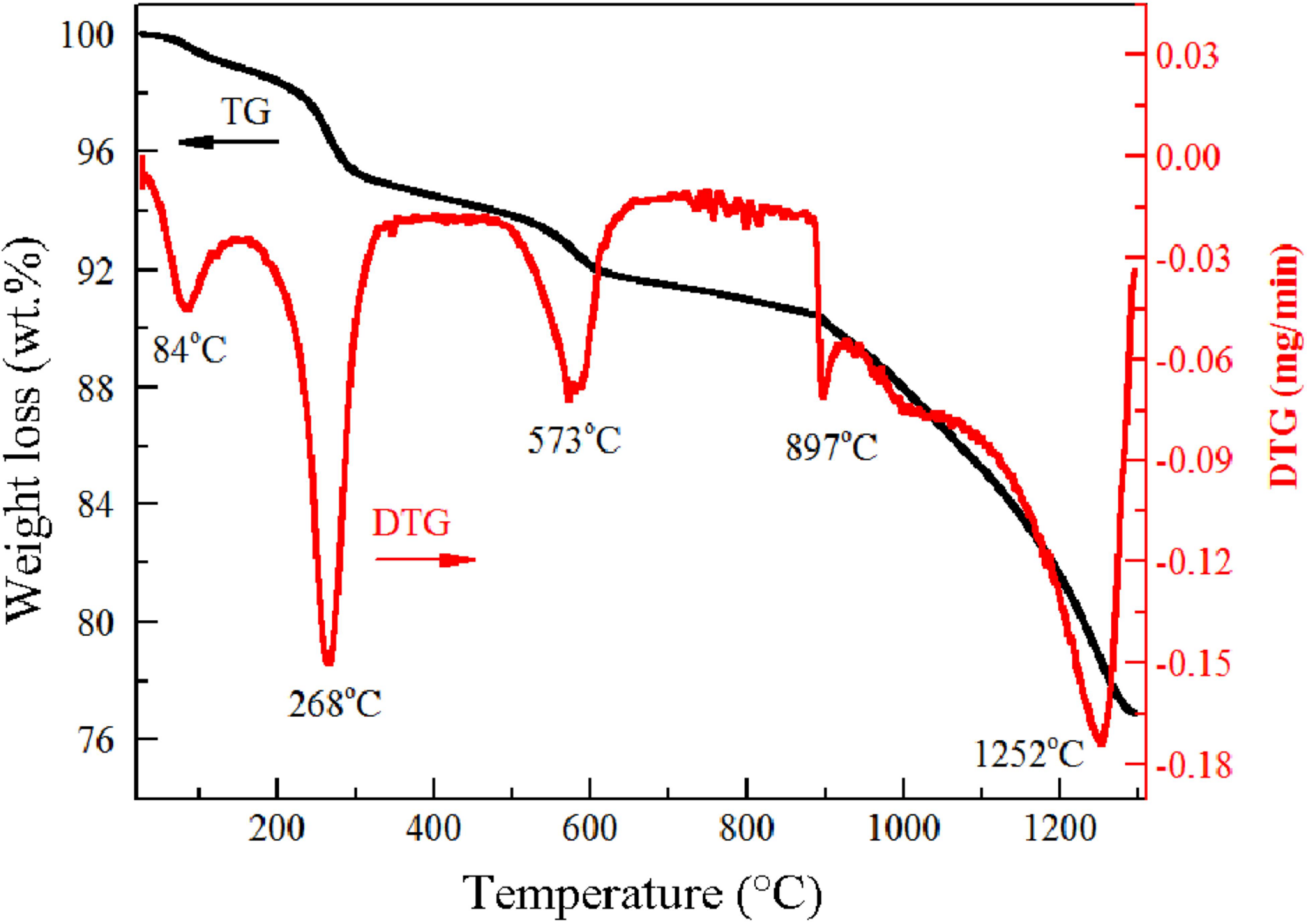
Differential thermal analysis–TGA curves for the Na_2_SO_4_/laterite blends.

Figs 1 and 2 show the TG and DTG curves of the decomposition of the laterite nickel ore and the Na_2_SO_4_/laterite blends, respectively. The weights of both samples decreased with increasing temperature.

In Fig 1, four obvious stages of mass loss were identified. The first stage reaction occurred between 34°C and 100°C and included the evaporation of the free water in the laterite. The second stage was between 238°C and 278°C and was caused by the transformation of goethite ores to iron oxide. The third stage involved the dehydroxylation of kaolinite and serpentine and occurred between 554°C and 602°C. The last stage occurred between 1100°C and 1145°C and involved only 3% of the total mass loss.

In Fig 2, five stages of mass loss were identified. Before reaching 863°C, the weight loss curves of the Na_2_SO_4_/laterite blends displayed almost the same trend as those of the laterite, which indicates that they had the same decomposition behavior. At temperatures of 987–1300°C, the TG curves of the Na_2_SO_4_/laterite blends decreased sharply compared to those of laterite, which is attributed to the decomposition of Na_2_SO_4_; Na_2_SO_4_ thus reacted with laterite in this temperature range.

Tables 2 and 3 show the parameters that were obtained from the decomposition experiments, including the initial decomposition temperature (T_I_), the final decomposition temperature (T_F_), the corresponding peak temperatures (T_P_), and the maximum weight loss rates (dW_i_/dt)_max_. From room temperature to 100°C, free water evaporated from the samples, which made the determination of T_I_ difficult [24,25]. Regular decomposition data cannot be obtained until 238°C; therefore, the initial temperature of decomposition T_I_ is defined as 238°C.

**Table 2:** Characteristic parameters of laterite decomposition (wt%).

Stage	Decomposition range (°C)		(dW_i_/dt)_max_ (min^-1^)	Peak temperature (°C)
	T_I_	T_F_		T_P_
a	238	278	-0.1684	268
b	554	602	-0.08106	587
c	1100	1145	-0.01893	1126

**Table 3:** Characteristic parameters of Na_2_SO_4_/laterite blend decomposition (wt%).

Stage	Decomposition range (°C)		(dW_i_/dt)_max_ (min^-1^)	Peak temperature (°C)
	T_I_	T_F_		T_P_
a	238	278	-0.1177	268
b	554	602	-0.0562	573
c	892	914	-0.0556	897
d	1241	1286	-0.1368	1252

At 600°C, the weight loss without Na_2_SO_4_ is 11.18 wt%, but with Na_2_SO_4_, it is 8.86 wt%. As Na_2_SO_4_ cannot be decomposed at 600°C and as the initial the initial Na_2_SO_4_ content of the Na_2_SO_4_/laterite blends was 20 wt% Na_2_SO_4_, the weight loss of the Na_2_SO_4_/laterite blends was due to the decomposition of laterite alone. Then, based on the weight of the 20 wt% Na_2_SO_4_, we can deduce that the weight loss is 11.08 wt%, which is similar to the weight loss without Na_2_SO_4_.

When the decomposition of the laterite nickel ore is complete at 1300°C, the volatile content is approximately 14.63 wt%. The decomposition of the Na_2_SO_4_/laterite blends produces approximately 23.12 wt% of volatiles under the same experimental conditions.

From room temperature to 890°C, the laterite and the Na_2_SO_4_/laterite blends have similar corresponding peak temperatures in their DTG curves. At temperatures higher than 897°C, the decomposition of laterite has only one peak temperature in its DTG curve at 1126°C, but the Na_2_SO_4_/laterite blends have two peak temperatures at 897°C and 1252°C.

### Kinetic characteristics

The values of *E* and *A* (pre-exponential factor) can be determined by Eq (3) and are shown in Tables 4 and 5, respectively.

**Table 4:** Kinetic parameters for laterite decomposition.

Stage	Temp (°C)	Conversion range (%)	E (kJ/mol)	A (min^-1^)	R^2^
a	238–278	25.88–42.43	87.13	2.83×10^5^	0.9947
b	554–602	70.44–81.18	123.63	9.75×10^4^	0.9927
c	1100–1145	94.77–97.21	145.66	2.82×10^5^	0.9999

**Table 5:** Kinetic parameters for the decomposition of Na_2_SO_4_/laterite blends.

Stage	Temp (°C)	Conversion range (%)	E (kJ/mol)	A (min^-1^)	R^2^
a	238–278	9.88–17.50	66.35	2.77×10^5^	0.9968
b	554–602	29.57–34.19	109.58	1.43×10^5^	0.9939
c	892–914	41.50–43.68	108.50	2.58×10^5^	0.9930
d	1241–1286	89.09–99.24	91.37	4.14×10^6^	0.9832

**Fig 3:**
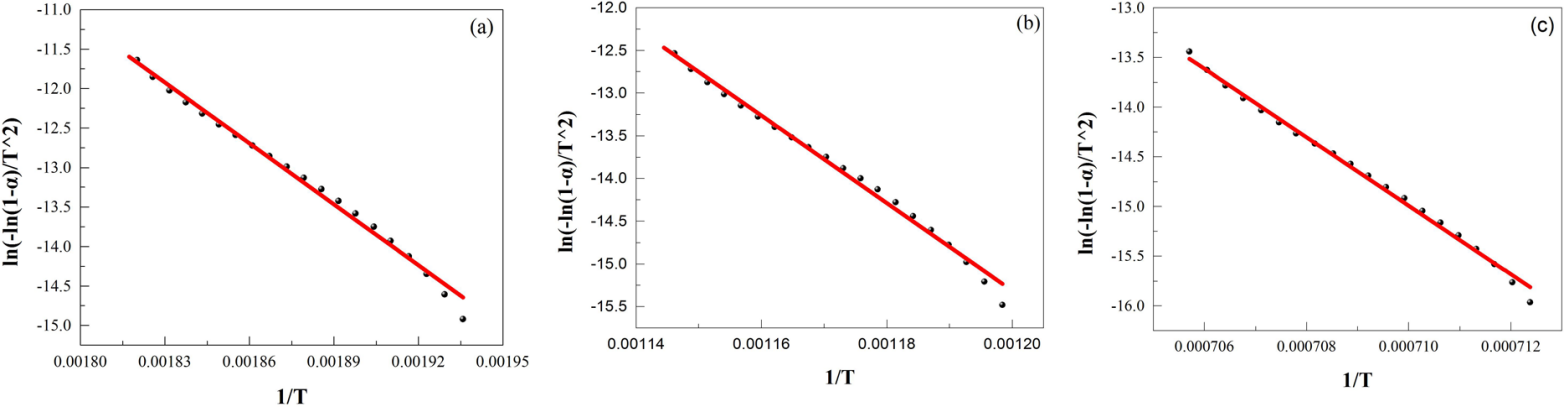
Plots of *ln(−ln(1−α)/T*^*2*^*)* vs. *1/T* for laterite decomposition recalculated by the multi-step integral method. (a), 238–278°C; (b), 554–602°C; (c), 1100–1145°C.

**Fig 4:**
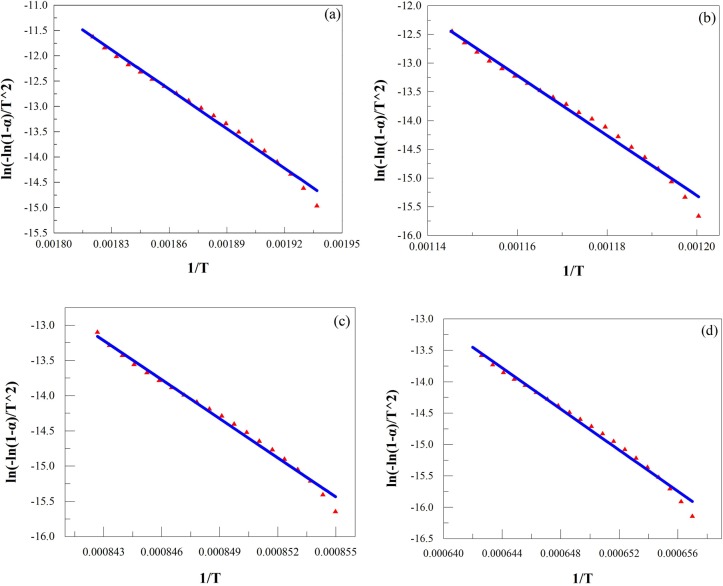
Plots of *ln(−ln(1−α)/T*^*2*^*)* vs. *1/T* for Na_2_SO_4_/laterite blend decomposition recalculated by the multi-step integral method. (a), 238–278°C; (b), 554–602°C; (c), 892–914°C; (d), 1241–1286°C.

Figs 3 and 4 show plots of *ln(−ln(1−α)/T*^*2*^*)* versus *1/T*, respectively. The plots for laterite are similar to those for the Na_2_SO_4_/laterite blends at temperatures between room temperature and 890°C. However, above 897°C, laterite behaves differently from the Na_2_SO_4_/laterite blends.

The kinetic parameters are calculated from the characteristic peaks, which were selected from Figs 1 and 2. Thus, these plots can be represented by the main decomposition region. From Figs 3 and 4, the plots of *ln(−ln(1−α)/T*^*2*^*)* versus *1/T*, 7 straight lines can be obtained, which indicated that the laterite decomposition and Na_2_SO_4_/laterite blend decomposition can be classified as a first order reaction. Therefore, the entire decomposition process can be described by one first-order reaction, which is consistent with the results from the literature [16–18].

The relative activation energies of the decomposition of laterite closely resemble those of the Na_2_SO_4_/laterite blends, which indicates that Na_2_SO_4_ did not change the decomposition of laterite between room temperature and 897°C. The melting point of Na_2_SO_4_ is 884°C [26], which is very close to 897°C. As the solid-state reaction requires more energy than the solid-liquid heterogeneous reactions [27], when the Na_2_SO_4_ begins to melt, it can accelerate the reaction of Na_2_SO_4_ with laterite. Therefore, the temperature of 897°C, which yields one of the fastest reaction rates, is very close to the melting point of Na_2_SO_4_.

However, above 897°C, the Na_2_SO_4_/laterite blends are different from laterite, which undergoes only one reaction with a relative activation energy of 145.66 kJ/mol. In the presence of Na_2_SO_4_, there are two reaction processes with relative activation energies of 108.50 and 91.37 kJ/mol.

Li et al. reported a lower melting point in the presence of Na_2_SO_4_, which could lead to the precipitation of larger particles [12]. Therefore, Na_2_SO_4_ significantly influences laterite decomposition through direct involvement and by changing the mechanism. Although adding Na_2_SO_4_ cannot change the reaction order, it can reduce the activation energy of laterite decomposition; thus, the reaction rate could be accelerated, and the reaction temperature could be markedly reduced.

### Decomposition mechanism

#### Laterite decomposition mechanism

To identify the mechanism of laterite decomposition, the laterite ores were roasted under a 100 ml/min Ar flow for 4 h at temperatures of 87°C, 268°C, 587°C, and 1126°C.

**Fig 5:**
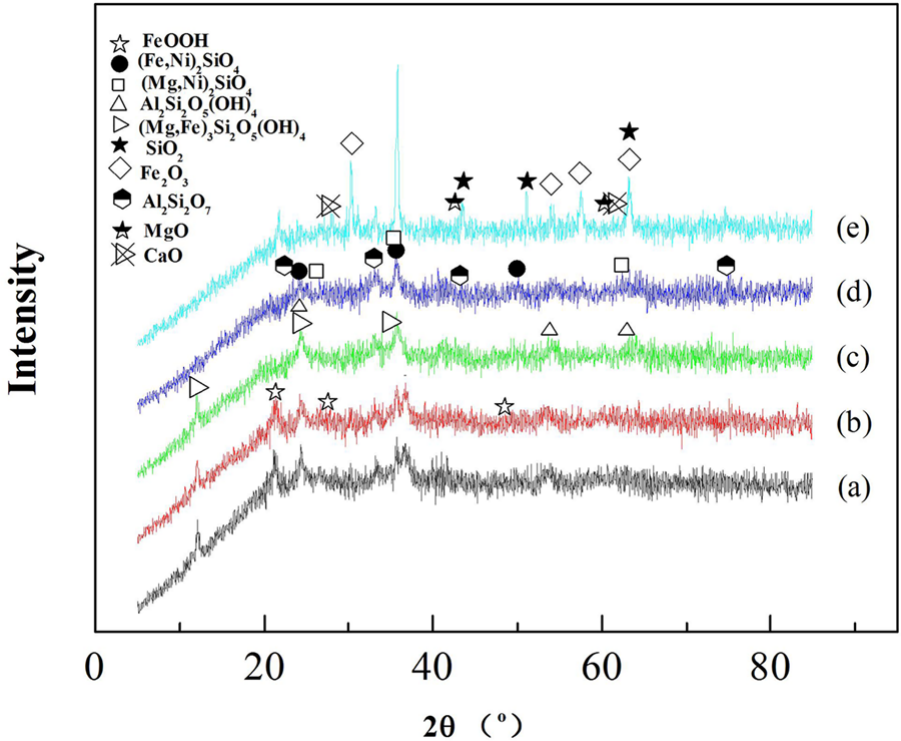
XRD patterns of laterite ores roasted under Ar at different temperatures. (a) raw ore, (b) 87°C, (c) 268°C, (d) 587°C, (e) 1126°C.

**Fig 6:**
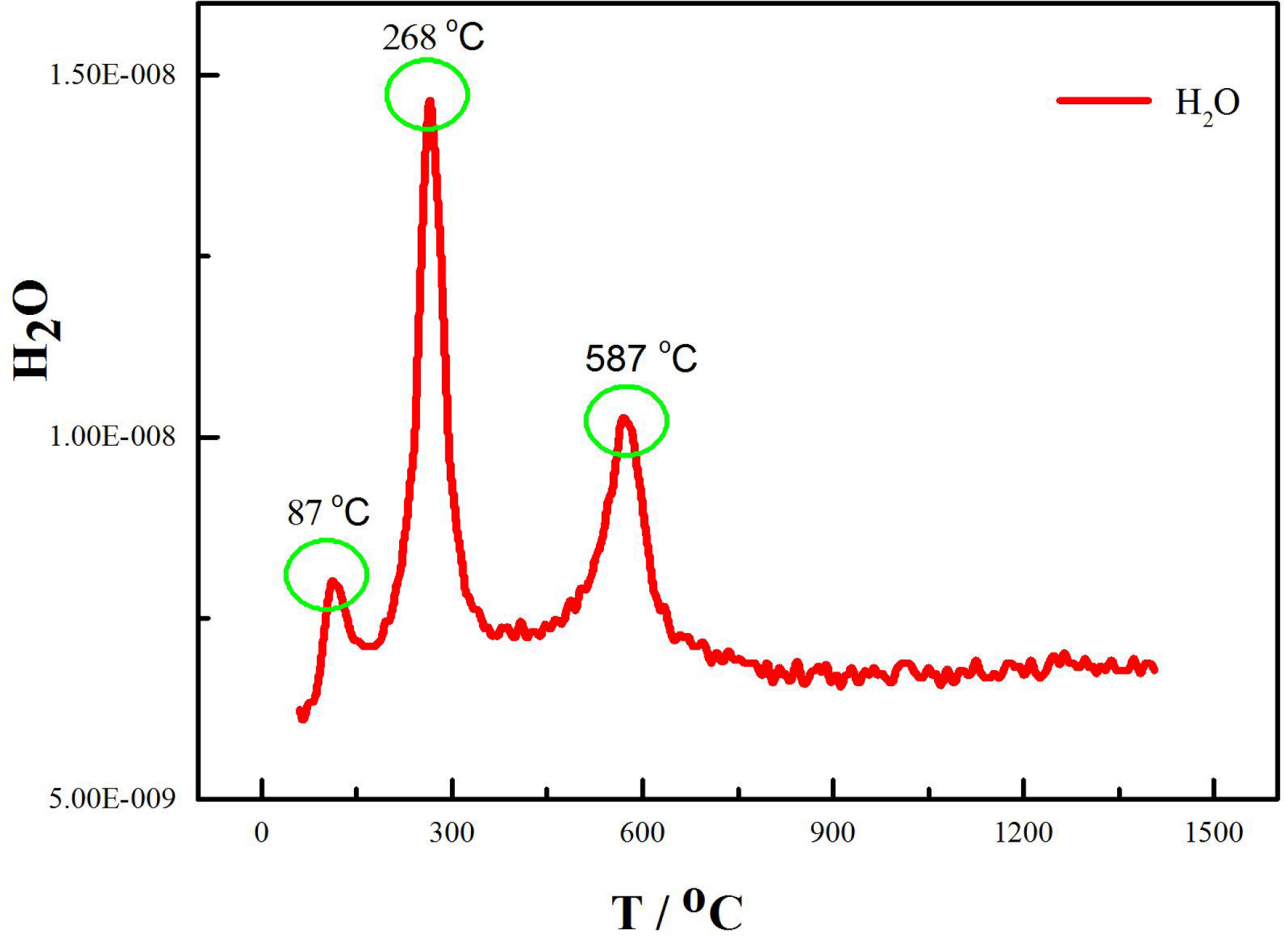
MS fragmentation intensities of H_2_O during the thermal decomposition of the laterite.

**Fig 7:**
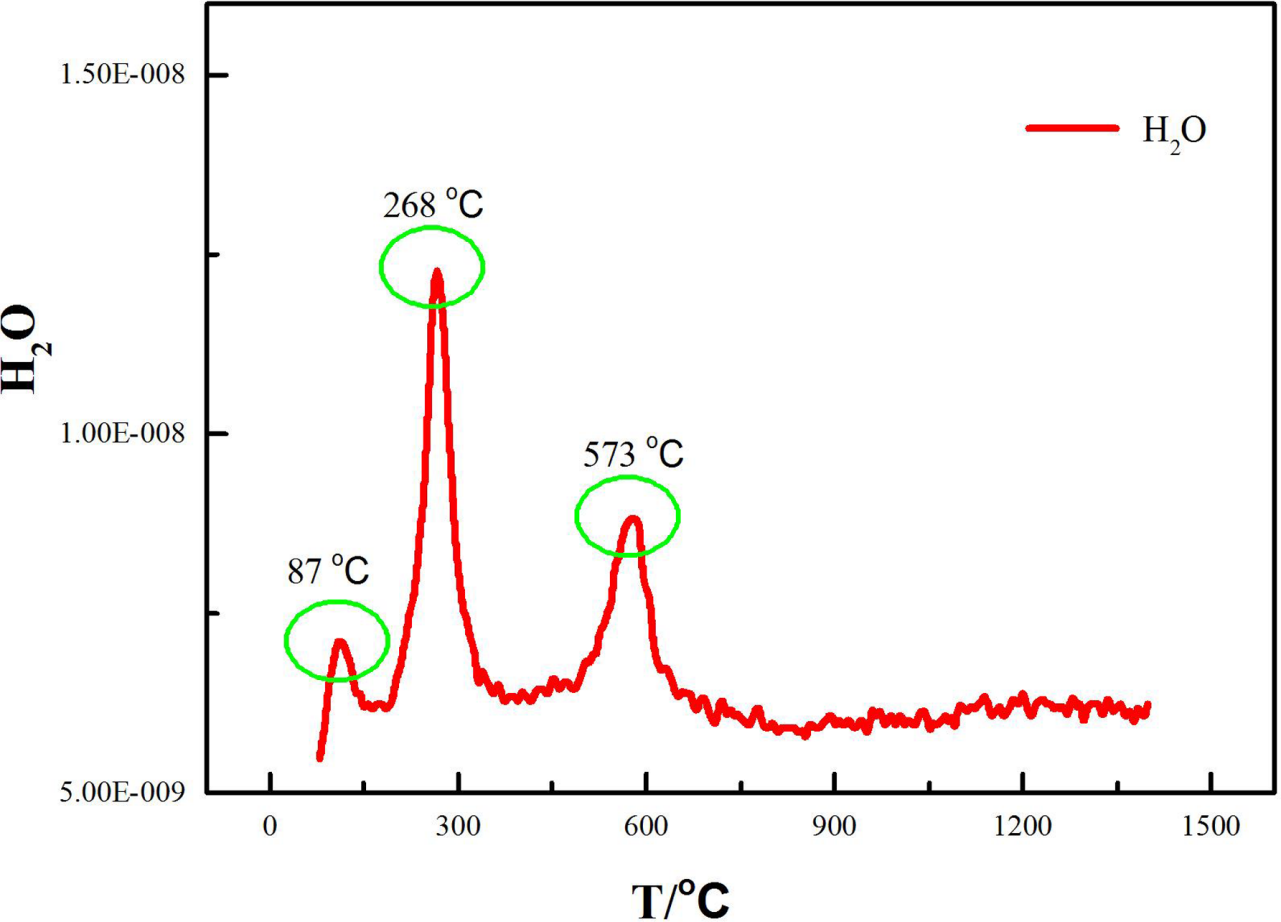
MS fragmentation intensities of H_2_O during the thermal decomposition of the Na_2_SO_4_/laterite blends.

The phase of the roasted ores can be elucidated from the XRD patterns (Fig 5), and the volatile component was analyzed by TG-MS. The variations in the H_2_O content during the thermal decomposition of laterite with and without the addition of Na_2_SO_4_ are shown in Figs 6 and 7, respectively.

Figs 1, 5 and 6 show that the decomposition process of laterite can be divided into four stages. The first stage (34°C<T<100°C) included the evaporation of the free water in the laterite. In the second stage (238°C<T<278°C), the goethite phase disappears after being roasted under Ar flow for 4 h (Fig 5B). Jang et al. investigated the transformation process of goethite ores to pure iron and found that water was released from the ores at low temperatures [28]. Our results show that water was the main volatile component in the second stage (Fig 6), which is consistent with results from the literature [28]. This process can be expressed by Eq (7):

$$2\alpha \text{-FeOOH}(\text{s})=\alpha {\text{-Fe}}_{2}{\text{O}}_{3}\left(\text{s}\right)+{\text{H}}_{2}\text{O}(\text{g})$$ (7)

As a result, new crystalline phases ((Fe,Ni)_2_SiO_4_, (Mg,Ni)SiO_3_, (Fe,Ni)_2_SiO_4_, (Fe,Ni)SiO_3_, SiO_2_, Al_2_Si_2_O_7_) formed (Fig 5C) during laterite decomposition at 587°C for 4 h, and the main volatile component was H_2_O (Fig 6). The water was produced by the dehydroxylation of kaolinite and serpentine at temperatures between 554°C and 602°C [29]. The process can be expressed by the following formulas:

$${2(\text{Mg},\text{Fe},\text{Ni})}_{3}{\text{Si}}_{2}{\text{O}}_{5}{\left(\text{OH}\right)}_{4}{=(\text{Mg},\text{Ni})}_{3}{\text{Si}}_{2}{\text{O}}_{5}{\left(\text{OH}\right)}_{4}{+(\text{Fe},\text{Ni})}_{3}{\text{Si}}_{2}{\text{O}}_{5}{\left(\text{OH}\right)}_{4}$$ (8)

$${2(\text{Mg},\text{Ni})}_{3}{\text{Si}}_{2}{\text{O}}_{5}{\left(\text{OH}\right)}_{4}{=(\text{Mg},\text{Ni})}_{2}{\text{SiO}}_{4}{+(\text{Mg},\text{Ni})\text{SiO}}_{3}{+2\text{SiO}}_{2}+{\text{H}}_{2}\text{O}$$ (9)

$${2(\text{Fe},\text{Ni})}_{3}{\text{Si}}_{2}{\text{O}}_{5}{\left(\text{OH}\right)}_{4}{=(\text{Fe},\text{Ni})}_{2}{\text{SiO}}_{4}{+(\text{Fe},\text{Ni})\text{SiO}}_{3}{+2\text{SiO}}_{2}+{\text{H}}_{2}\text{O}$$ (10)

$${\text{Al}}_{2}{\text{Si}}_{2}{\text{O}}_{5}{\left(\text{OH}\right)}_{4}{=\text{Al}}_{2}{\text{Si}}_{2}{\text{O}}_{7}{+2\text{H}}_{2}\text{O}$$ (11)

**Fig 8:**
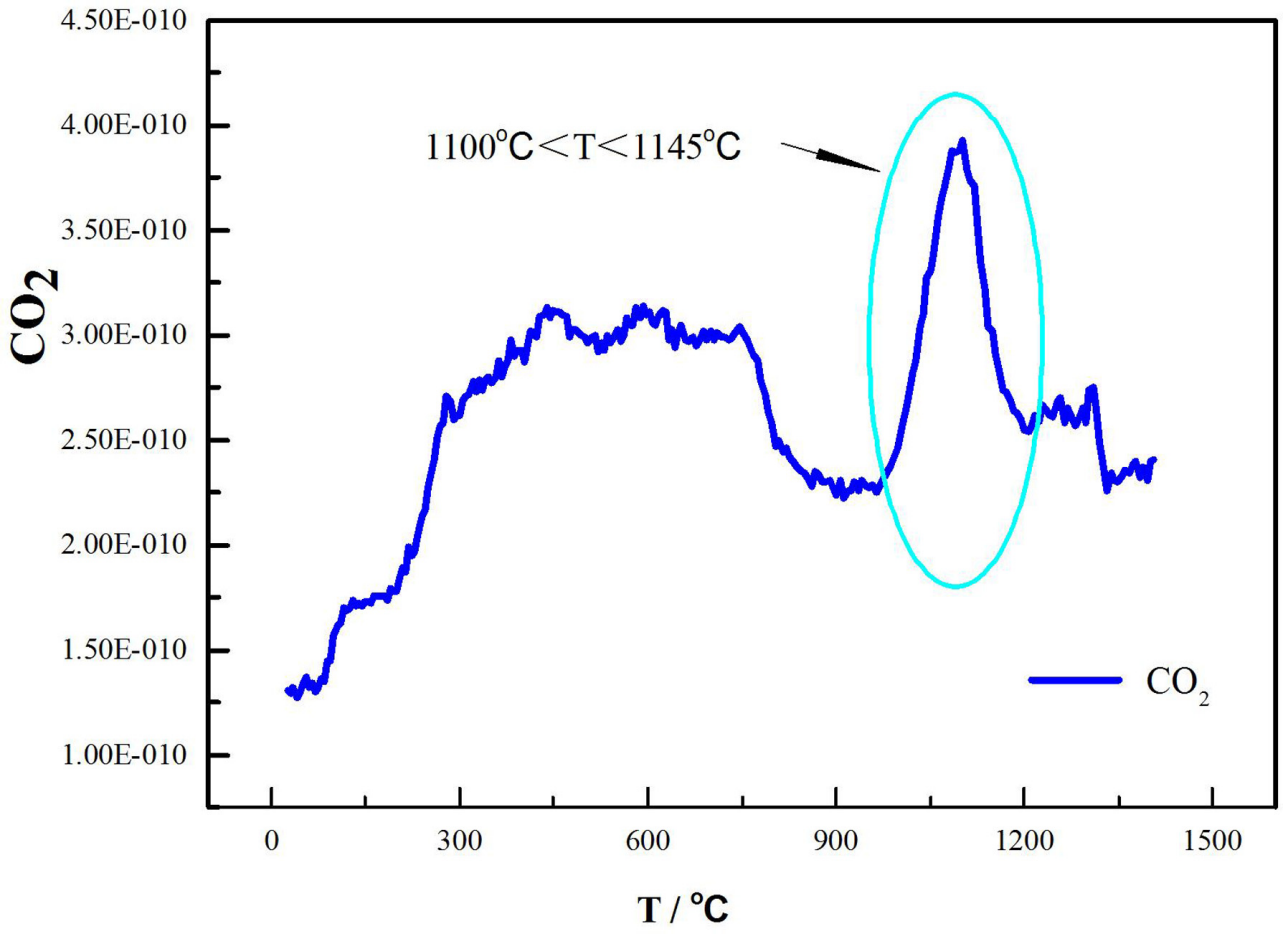
MS fragmentation intensities of CO_2_ during the thermal decomposition of laterite.

The mechanism of laterite decomposition in the fourth stage (1100°C<T<1145°C) has not been determined in the literature. MS was used to identify the main volatile component in this temperature range, and the roasted sample, which was roasted under Ar flow for 4 h at 1126°C, was analyzed with XRD to identify the main component of the decomposition of the laterite. Fig 5D shows that CaO and MgO were identified as the new phases. CO_2_ was determined to be the main volatile component by MS at temperatures between 1100 and 1145°C (Fig 8). Dolomite is a component of laterite [4]. Yoshida et al. investigated the decomposition of dolomite ores to CaO and MgO and found that CO_2_ was released from the ores [30], which is consistent with our observations. However, our results partially contradict those reported by Conesa et al., who found that the primary decomposition temperature of dolomite was 780–800°C during the gasification and pyrolysis of *Posidonia oceanica* in the presence of dolomite [31]. Presumably, this difference was caused by the dispersion of dolomite in the laterite and the interaction with the laterite complex. The reaction [31] in the fourth stage of our study can be expressed by

$${\text{Ca},\text{Mg}(\text{CO}}_{3}{)}_{2}\overset{1126{}^{\circ}\text{C}}{\to }{\text{CaCO}}_{3}{+\text{MgCO}}_{3}$$ (12)

$${\text{Ca},\text{Mg}{(\text{CO}}_{3})}_{2}\overset{1126{}^{\circ}\text{C}}{\to }{\text{CaO}+\text{MgO}+2\text{CO}}_{2}$$ (13)

At temperatures between room temperature and 890°C, water was the product of laterite decomposition, which caused approximately 92% of the total mass loss. At temperatures above 890°C, the main volatile component was CO_2_, which caused only 3% of the total mass loss.

### Decomposition mechanism of the Na_2_SO_4_/laterite blends

To identify the mechanism of decomposition of the Na_2_SO_4_/laterite blends, the samples of the Na_2_SO_4_/laterite blend ores were roasted under a 100 ml/min Ar flow for 4 h at temperatures of 87°C, 268°C, 573°C, 897°C, and 1252°C.

**Fig 9:**
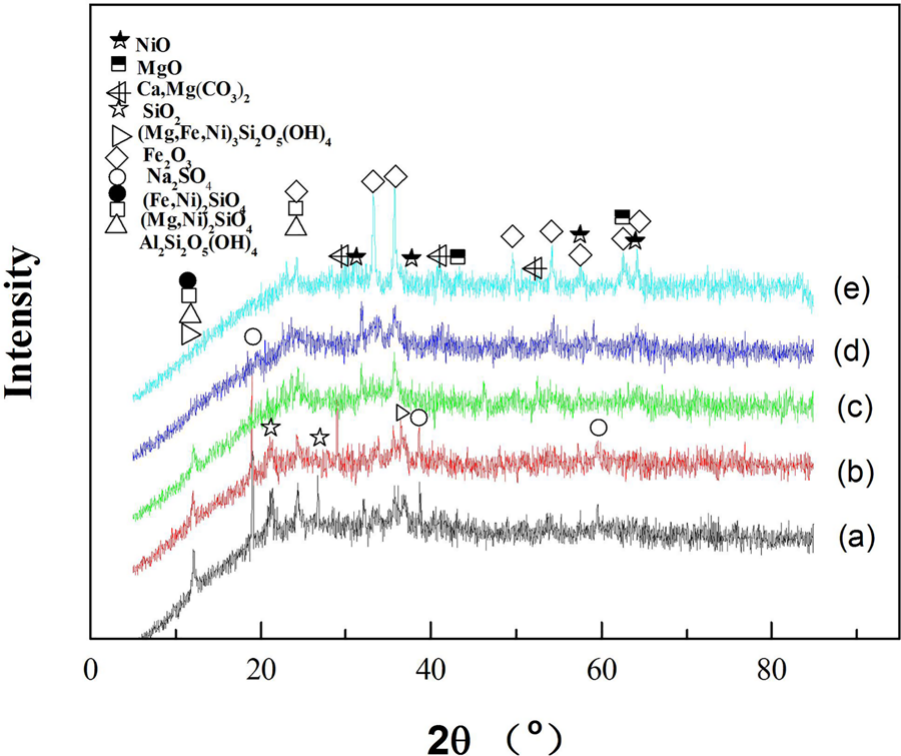
XRD patterns of the Na_2_SO_4_/laterite blends roasted under Ar at different temperatures. (a) raw ore, (b) 84°C, (c) 268°C, (d) 573°C, (e) 897°C.

Fig 9 shows the phase of the roasted ores, which were roasted under a 100 ml/min Ar flow for 4 h at temperatures of 84°C, 268°C, 573°C, and 897°C, as elucidated from the XRD patterns. The volatile component was analyzed by TG-MS. Fig 7 shows the variation in the H_2_O content during the thermal decomposition of the Na_2_SO_4_/laterite blends.

Figs 2, 7 and 9 show that the decomposition of the Na_2_SO_4_/laterite blends can be divided into five stages. The decomposition ranges of the Na_2_SO_4_/laterite blends are similar to those of the laterite in the first three stages, and H_2_O is the volatile component. Therefore, the first three stages are similar to the decomposition process of the laterite ores, which suggests that Na_2_SO_4_ does not affect laterite decomposition below 700°C. Increasing the roasting temperature to 892°C results in a sharp decrease in the amounts of the Na_2_SO_4_/laterite blends; a weight loss of approximately 14 wt% occurred between 897°C and 1300°C, where the laterite had experienced a weight loss of approximately 1 wt%. It can be inferred that Na_2_SO_4_ has a significant impact on the decomposition of laterite at temperatures above 897°C.

**Fig 10:**
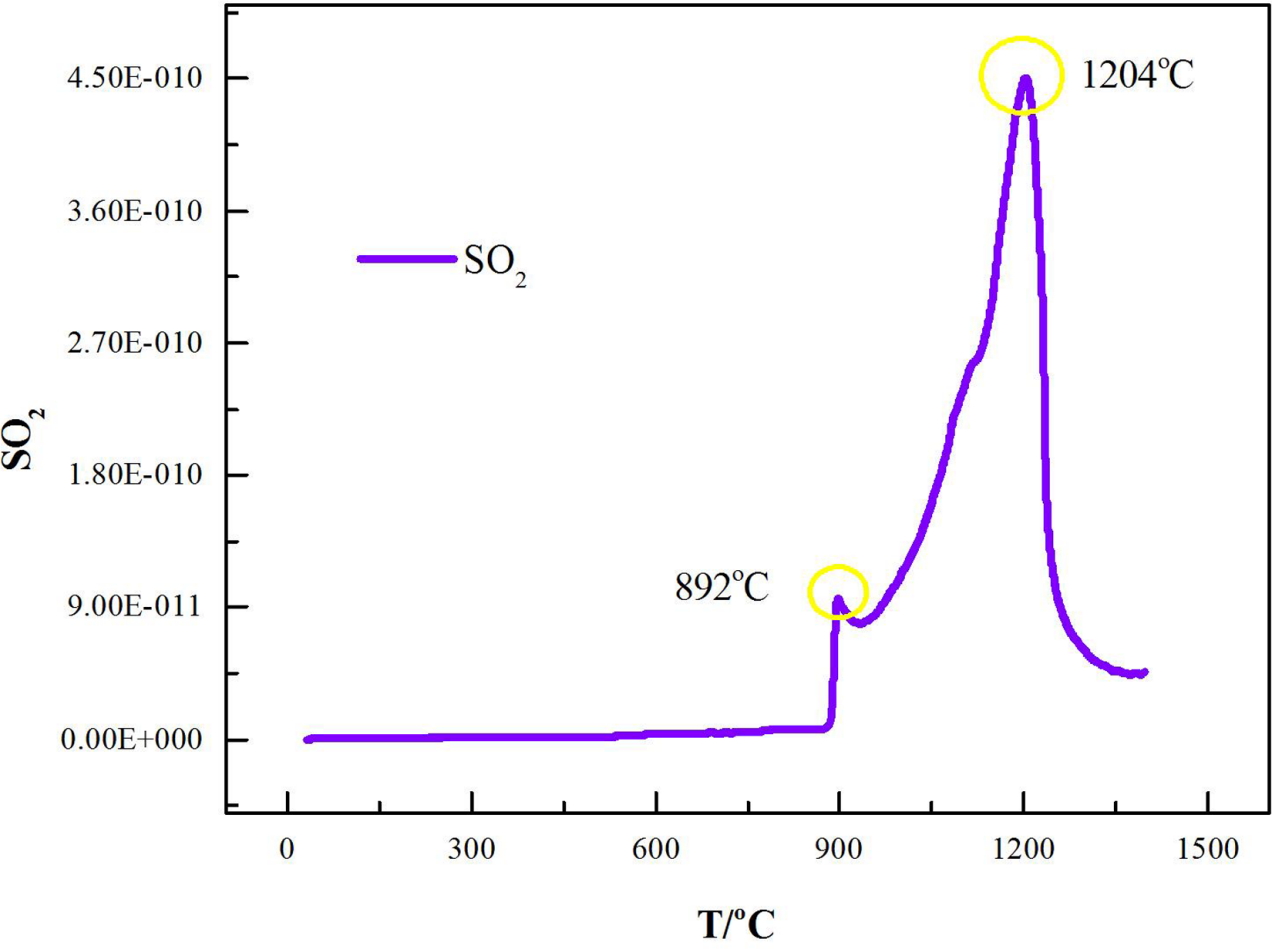
MS fragmentation intensities of SO_2_ during thermal decomposition of the Na_2_SO_4_/laterite blends.

**Fig 11:**
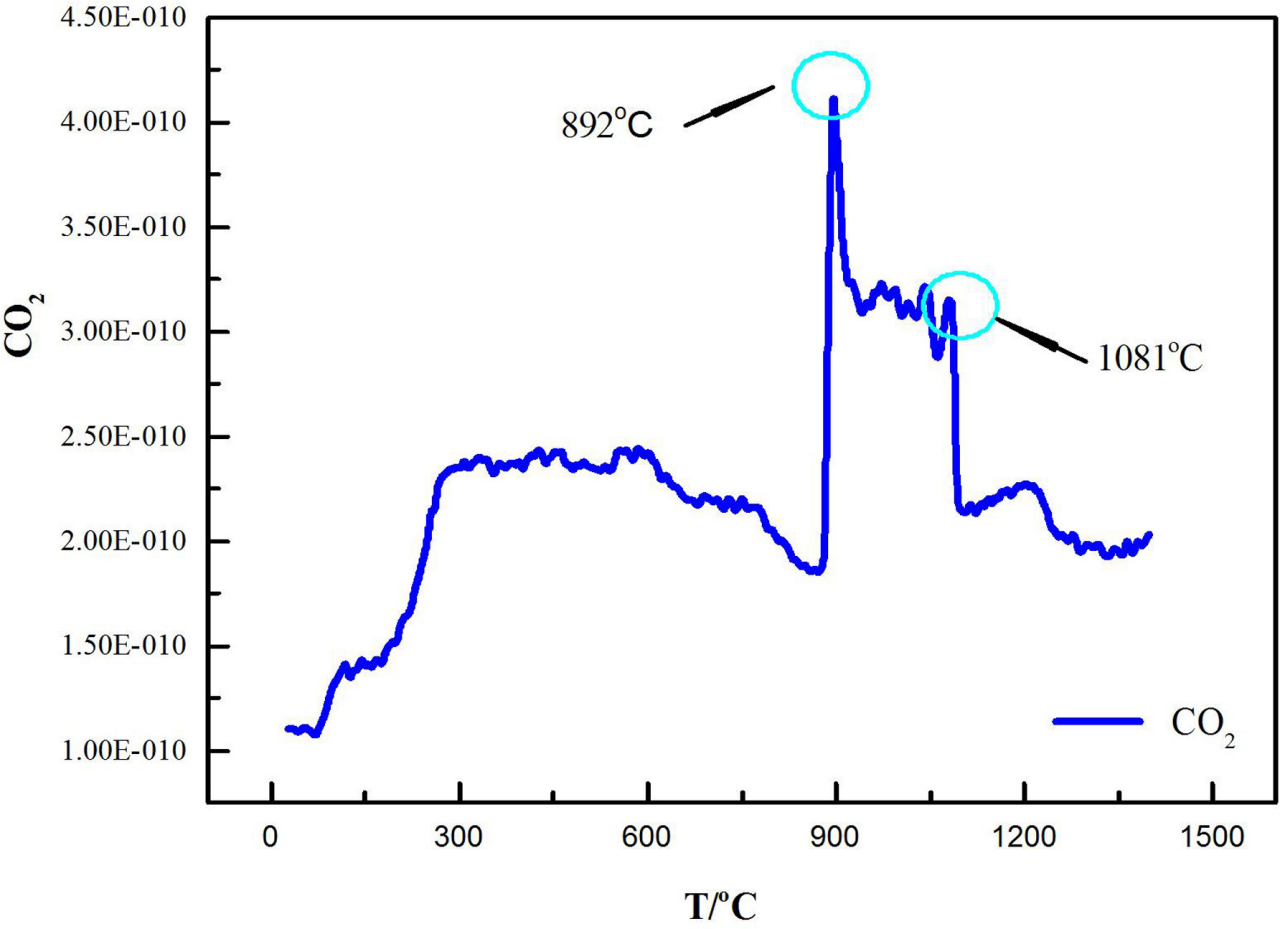
MS fragmentation intensities of CO_2_ during thermal decomposition of the Na_2_SO_4_/laterite blends.

To determine whether the weight loss was caused by the Na_2_SO_4_ decomposition itself, pure Na_2_SO_4_ was heated under a 100 ml/min Ar flow from room temperature to 1300°C by TG-MS. Reagent-grade Na_2_SO_4_ was pyrogenated via thermogravimetric analysis, and no significant decomposition of Na_2_SO_4_ was observed. The result indicates that the mass loss of the Na_2_SO_4_/laterite blends cannot be due to Na_2_SO_4_ decomposition alone. Freyer et al. investigated the phase diagram of the Na_2_SO_4_—CaSO_4_ system and found that Na_2_SO_4_ cannot be decomposed until 1404°C [32], which indicates that the mass loss of Na_2_SO_4_/laterite blends cannot be caused by only Na_2_SO_4_ decomposition. In addition, SO_2_ was detected by MS (Fig 10) along with the emitted CO_2_ beginning at 892°C. Based on the MS fragmentation intensities of CO_2_ during the thermal decomposition of the Na_2_SO_4_/laterite blends (Fig 11), the initial temperature of CO_2_ emission is 897°C, which is 200°C lower than the decomposition temperature of the laterite.

The roasted products of the Na_2_SO_4_/laterite blends, which were roasted at 897°C for 4 h, were detected by XRD. CaO and MgO were found in the roasted residue (Fig 9). Gunasekaran et al. investigated the thermal decomposition of natural dolomite and found that the decomposition temperature of dolomite is 600–850°C [33], which indicates that in the presence of Na_2_SO_4_, the decomposition temperature of dolomite can be reduced to nearly the decomposition temperature of natural dolomite. Lu et al. investigated the reduction of prepared nickel laterite ore by H_2_. The experiments were conducted at 800°C with the addition of 20 wt% Na_2_SO_4_ and obtained a maximum nickel content of 5.63% and a nickel recovery of 83.59% [9], which indicates that the pyrometallurgical operating temperature can be reduced to a range of 892–1080°C in the presence of Na_2_SO_4_, which is approximately 200°C lower than in the conventional process [7]. Therefore, in the fourth stage (897°C<T<914°C), it can be inferred that dolomite can be decomposed in the presence of the Na_2_SO_4_ in this temperature range. The process is described in the literature [9,32] and can be expressed as Eq (12) and Eq (13).

Nevertheless, it is worth investigating whether the CaO, which decomposed from the dolomite, can affect the emission of SO_2_, as the CaO can react with SO_2_ to form CaSO_3_. Cubicciotti et al. investigated the thermal decomposition of CaSO_3_ and its enthalpy of formation and found that CaSO_3_ decomposed in the temperature range of 500–550°C [34]. Therefore, CaSO_3_ could not stably exist between 897°C and 914°C. In addition, the content of dolomite in the laterite ores was low and the amount of CaO released would be low. Therefore, the presence of CaO would have little influence on the emission of CO_2_ and SO_2_.

In the last stage (1241°C<T<1286°C), CO_2_ and SO_2_ were the main volatiles of the decomposition of the Na_2_SO_4_/laterite blends (Fig 10). Based on the products of the fourth stage of the decomposition of the Na_2_SO_4_/laterite blends, CO_2_ may be generated by the decomposition of the dolomite. Lu et al. investigated the standard Gibbs free energy and temperature diagram and found that SO_2_ was released from the ores at temperatures above 727°C [9]. Our results show that Na_2_SO_4_ reacted with laterite and emitted SO_2_, which was consistent with results that have been reported in the literature [9].

**Fig 12:**
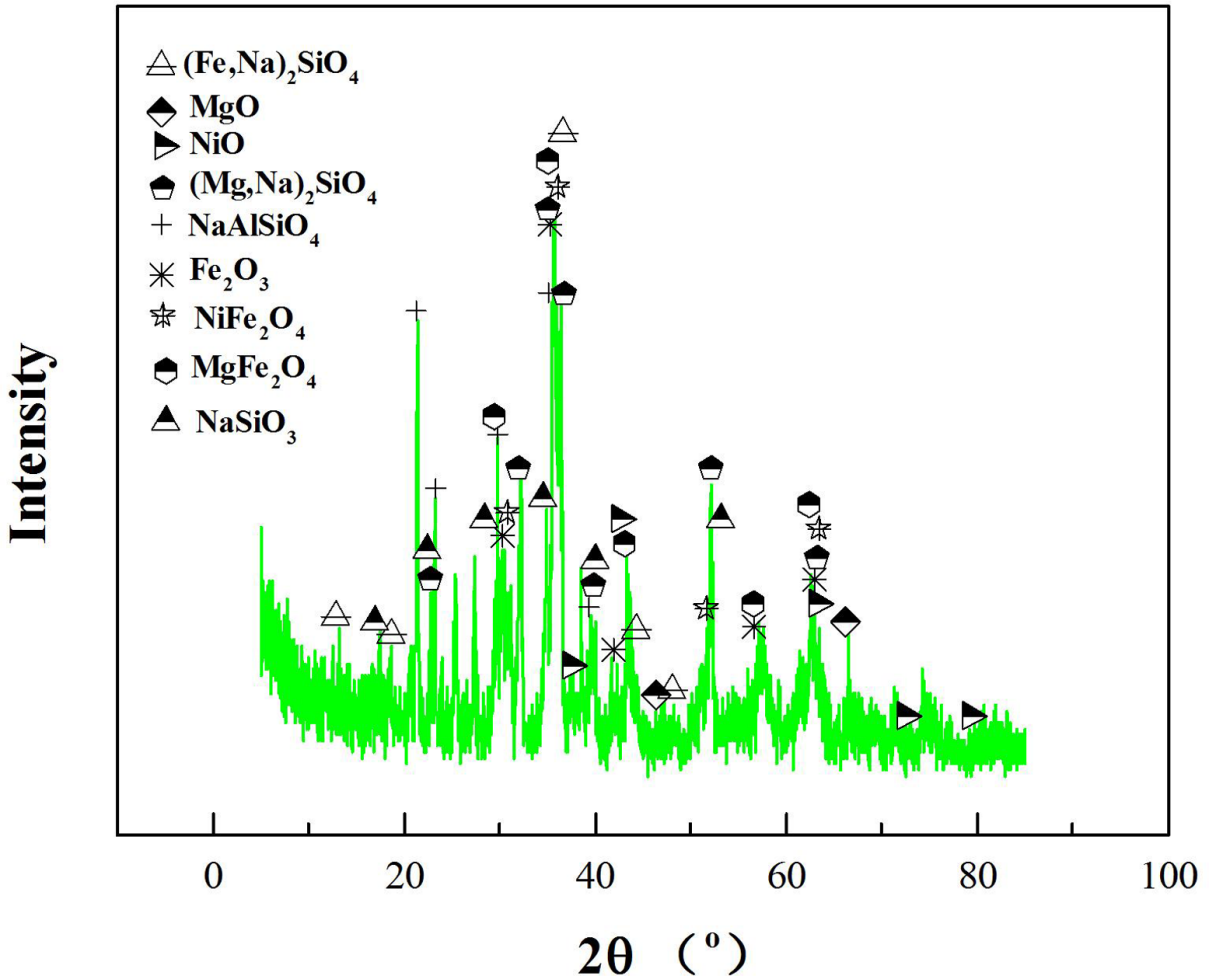
XRD patterns of the Na_2_SO_4_/laterite blends roasted under Ar at 1252°C for 4 h.

To determine what occurs when Na_2_SO_4_ reacts with laterite, the decomposition products, which were roasted at 1252°C for 4 h, were analyzed by XRD (Fig 12). (Mg,Ni)_2_SiO_4_, (Mg,Ni)_2_SiO_4_, (Fe,Ni)_2_SiO_4_ and (Fe,Ni)_2_SiO_4_ disappeared (Fig 9D), and several new phases formed, such as (Mg,Na)_2_SiO_4_, Na_2_SiO_3_, (Mg,Na)_2_SiO_4_ and Na_2_SiO_3_. Li et al. investigated the purification of nickeliferous laterite by reduction roasting in the presence of sodium sulfate and found that sodium sulfate can liberate Fe and Ni from lizardite and leave behind an Mg-rich olivine phase [12]. The results of this study show that the main residue of the Na_2_SO_4_/laterite blends under 1252°C in the last stage were consistent with those that have been reported in the literature [12]. Therefore, Na_2_SO_4_ reacted with (Mg,Ni)_2_SiO_4_ and (Mg,Ni)_2_SiO_4_, and a considerable amount of Na^+^ then replaced Ni^2+^ in (Mg,Ni)_2_SiO_4_ and (Mg,Ni)_2_SiO_4_. Meanwhile, the free Ni^2+^ formed NiO, which is beneficial for the ensuing nickel enrichment.

Mobin et al. found that transition-metal carbides interact with Na_2_SO_4_ to form a soluble sodium metal oxide or a metal sulfide depending upon the local conditions during the high temperature reaction [35]. Our research shows that the Na_2_SO_4_/laterite blends reach the molten state after roasting at 1252°C for 4 h, which is consistent with results from the literature [12].

Alkali ions preferentially take up next-nearest neighbor positions with respect to tetrahedral Fe^3+^ ions [36]. In our study, the Na^+^ replaced Fe^3+^ and Mg^2+^ in (Mg,Ni)_2_SiO_4_ and (Mg,Ni)_2_SiO_4_ to form Na_2_SiO_3_. Composite materials with mixed spinel nickel ferrite–barium titanate as co-existing phases can be synthesized [37]. Therefore, the Fe_2_O_3_, NiO and MgO that are released from (Mg,Ni)_2_SiO_4_ and (Mg,Ni)_2_SiO_4_ then react and form MgFe_2_O_4_ and NiFe_2_O_4_ (Eqs 14, 19 and 20). At the same time, Na^+^ replaces some of the Al^3+^ in the Al_2_Si_2_O_7_ and forms NaAlSiO_4_ (Eq 17). O’Neill et al. experimentally investigated the effect of the melt composition on trace element partitioning based on the activity coefficients of FeO, NiO, CoO, MoO_2_ and MoO_3_ in silicate melts and found that the activity coefficients of FeO, NiO and CoO vary by a factor of two over the same range of melt compositions [38]. In this study, we found that all of the materials combine to form a hard low-melting-point amorphous substance at high temperature with some alkaline metals. In the last stage of the laterite with and without the addition of Na_2_SO_4_, this mechanism is supported by the phase composition of the decomposition products (Fig 12) and the MS fragmentation intensities of SO_2_ during the decomposition at temperatures between 1241 and 1286°C (Fig 10). Therefore, the mechanism of laterite decomposition by Na_2_SO_4_ involved reacting some of the Na_2_SO_4_ with the calcined laterite ores, which resulted in laterite decomposition and the release of Ni^2+^ from the laterite and is beneficial for nickel enrichment. This process can be expressed as follows:

$${\left(\text{Mg},\text{Ni}\right)}_{2}{\text{SiO}}_{4}{+\text{Na}}_{2}{\text{SO}}_{4}{=(\text{Mg},\text{Na})}_{2}{\text{SiO}}_{4}{+2\text{NiO}+\text{SO}}_{2}(\text{g})$$ (14)

$${\left(\text{Mg},\text{Ni}\right)}_{2}{\text{SiO}}_{3}{+\text{Na}}_{2}{\text{SO}}_{4}{=\text{Na}}_{2}{\text{SiO}}_{3}{+2\text{MgO}+2\text{NiO}+\text{SO}}_{2}(\text{g})$$ (15)

$${\left(\text{Fe},\text{Ni}\right)}_{2}{\text{SiO}}_{4}{+\text{Na}}_{2}{\text{SO}}_{4}{=(\text{Fe},\text{Na})}_{2}{\text{SiO}}_{4}{+2\text{NiO}+\text{SO}}_{2}(\text{g})$$ (16)

$${\left(\text{Fe},\text{Ni}\right)}_{2}{\text{SiO}}_{3}{+\text{Na}}_{2}{\text{SO}}_{4}{=\text{Na}}_{2}{\text{SiO}}_{3}{+2\text{Fe}}_{2}{\text{O}}_{3}{+2\text{NiO}+\text{SO}}_{2}(\text{g})$$ (17)

$${\text{Al}}_{2}{\text{Si}}_{2}{\text{O}}_{7}{+\text{Na}}_{2}{\text{SO}}_{4}{=2\text{NaAlSiO}}_{4}{+\text{SO}}_{2}(\text{g})$$ (18)

$${\text{Fe}}_{2}{\text{O}}_{3}{+\text{MgO}=\text{MgFe}}_{2}{\text{O}}_{4}$$ (19)

$${\text{Fe}}_{2}{\text{O}}_{3}{+\text{NiO}=\text{NiFe}}_{2}{\text{O}}_{4}$$ (20)

### Reduction results

#### Reduction roasting-magnetic separation of the laterite with/without Na_2_SO_4_

**Fig 13:**
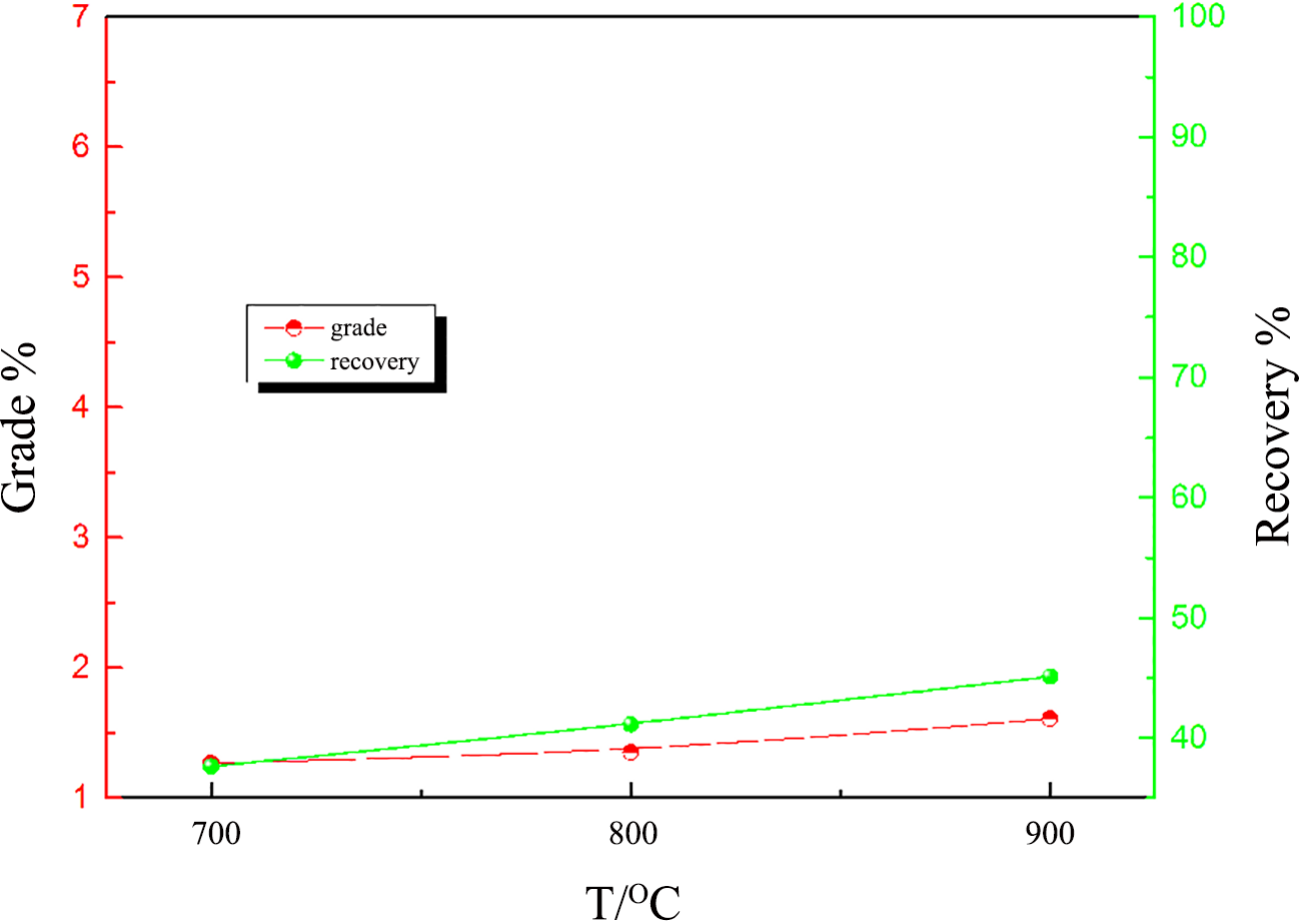
The effect of the reduction temperature on nickel–iron beneficiation (reduced for 120 min).

In the absence of Na_2_SO_4_, hydrogen reduction roasting-magnetic separation was conducted at 700, 800 and 900°C. The other experimental conditions were fixed, including the reducing time of 120 min, the gas rate of 2.7 L/min (H_2_:70%, N_2_:30%), grinding fineness of 90 wt% passing 0.043 mm and magnetic field intensity of 0.1 T. Fig 13 shows that the grade and recovery of Ni increases with increasing roasting temperature from 700 to 900°C. However, only a maximum of 1.61% Ni grade with recovery of 45.09% Ni is achieved at 900°C.

**Fig 14:**
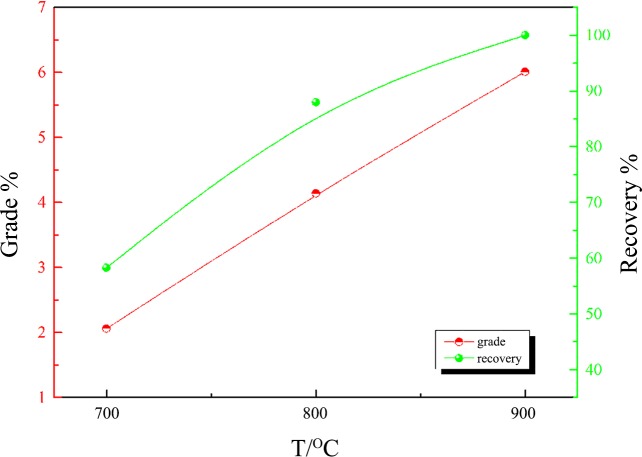
The effect of the reduction temperature on nickel–iron beneficiation (reduced for 120 min in the presence of 20 wt% sodium sulphate).

When the nickel laterite ore with the addition of 20 wt% Na_2_SO_4_ was reduced at 700, 800 and 900°C for 120 min, the Ni grade increased from 2.06% to 6.01% and the recovery of Ni increased from 58.29% to 100% from 700°C to 900°C, as shown in Fig 14. The roasting reduction temperature of the commercially existing pyrometallurgical process for nickel from nickel laterite is 1250–1400°C [12]. Therefore, in terms of operating temperature, the reduction in operating temperature to 900°C in the current work is an advantage.

#### SEM analysis

**Fig 15:**
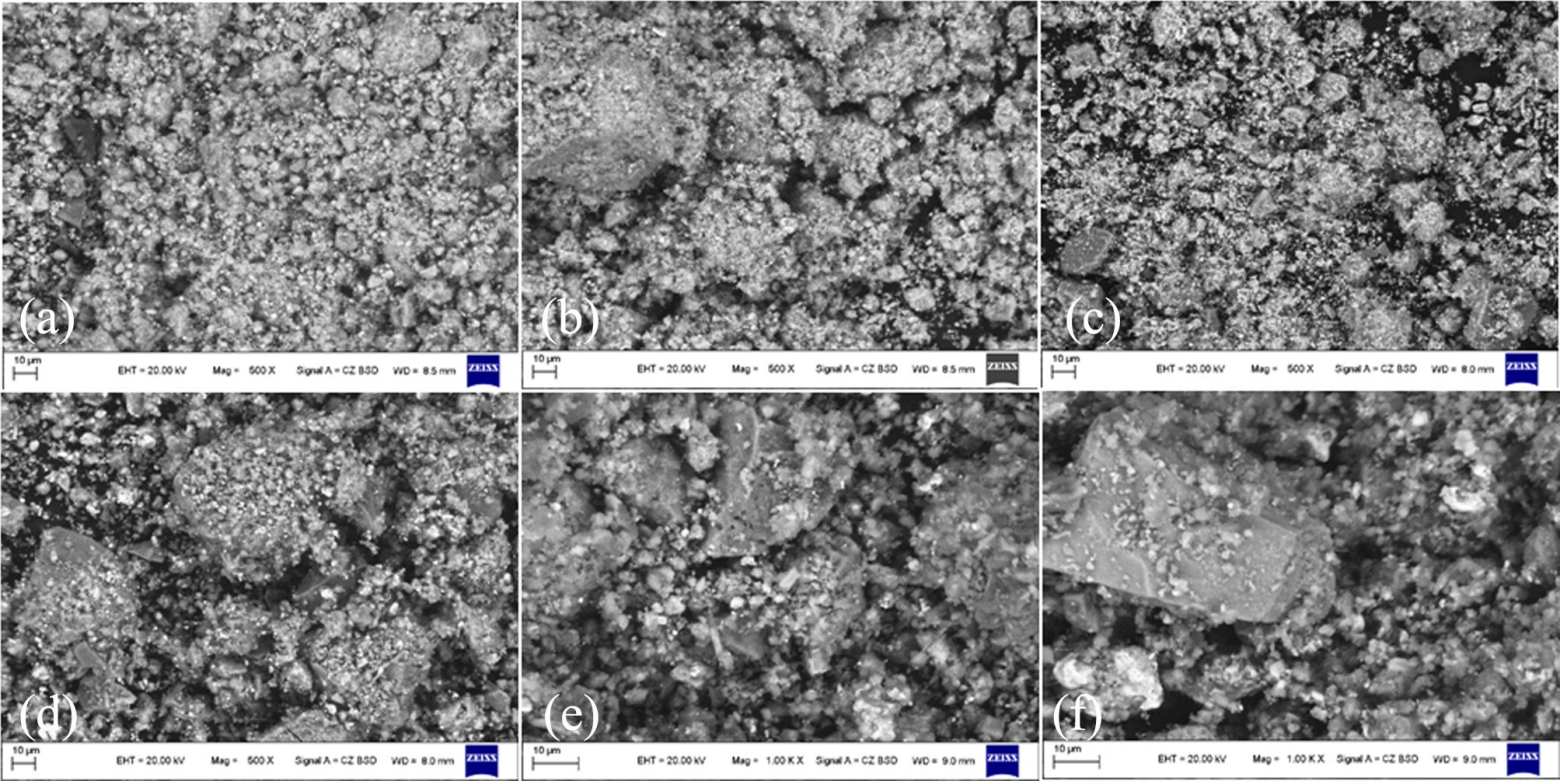
SEM images (backscattered electron images) of the roasted ores. (a)-(c) General overview of microstructure of mineral particles and the roasted ores without Na_2_SO_4_ at 700°C, 800°C, and 900°C, respectively. (d)-(f) General overview of microstructure of mineral particles and the roasted ores with 20 wt% Na_2_SO_4_ at 700°C, 800°C, and 900°C, respectively.

To reveal the effects of the Na_2_SO_4_ on the beneficiation of laterite ore, the reduced products were analyzed by SEM. The microstructure of the roasted ore (with/without Na_2_SO_4_) at different temperatures are shown in Fig 15.

Fig 15(A)–15(C) show the general microstructure of the roasted ores of laterite. The structure of the roasted ore is loose with a dispersed metallic mineral distribution. Furthermore, the roasted ore of Na_2_SO_4_/laterite blends exhibits a saponaceous surface and a compact structure as shown in Fig 15D–15F, which indicates that the roasted ore is molten with Na_2_SO_4_ during the reduction process [13]. The molten phase is beneficiated to achieve rapid grain coarsening and a higher concentration of Ni [9–12].

## Conclusions

The results of this study can be summarized as follows:

1. The decomposition of laterite can be divided into four stages: water evaporation, goethite decomposition, kaolinite and serpentine dehydroxylation, and dolomite decomposition with CO_2_ as the main volatile component. The activation energies for the different stages were 45.64 kJ/mol, 87.13 kJ/mol, 123.63 kJ/mol and 145.66 kJ/mol, respectively.
2. The decomposition of Na_2_SO_4_/laterite blends can be divided into five stages. The decomposition process is similar to that of laterite blends at 700°C. However, dolomite decomposed at 200°C lower than the temperature at which laterite decomposed in the fourth stage, and the activation energy was 108.50 kJ/mol. In the final stage, laterite decomposed in the presence of Na_2_SO_4_ and emitted SO_2_ with an activation energy of 91.37 kJ/mol.
3. Kinetic analyses revealed that the decomposition of laterite with and without the addition of Na_2_SO_4_ in an argon atmosphere can be described by one first-order reaction, However, Na_2_SO_4_ significantly influences laterite decomposition through direct involvement and can reduce the activation energy of laterite decomposition; thus, the reaction rate can be accelerated, and the reaction temperature can be markedly reduced.
4. The mechanism of the decomposition of Na_2_SO_4_/laterite blends may involve the reaction of Na_2_SO_4_ with laterite and the consequent decrease in the laterite decomposition temperature. Na^+^ was then replaced with Ni^2+^, which is an isomorphic host in the lattice of (Ni,Mg)_2_SiO_4_. Ni^2+^ was released and reacted with O^2-^ to form NiO, which facilitated nickel enrichment through the ensuing reduction. At the same time, Na_2_SO_4_ reacted with Mg_2_SiO_4_ and Fe_2_SiO_4_ to form low-melting-point compounds.
5. The roasted ore with the addition of Na_2_SO_4_ exhibits a compact structure and can be formed in the molten phase with Na_2_SO_4_ during the reduction process. The molten phase is beneficiated to achieve rapid grain coarsening and a higher concentration of Ni. Moreover, the pyrometallurgical operating temperature can be reduced to a range of 892–1080°C in the presence of Na_2_SO_4_, which is approximately 200°C lower than in the conventional process.
